# Association of obesity, diabetes, and hypertension with arsenic in drinking water in the Comarca Lagunera province (north-central Mexico)

**DOI:** 10.1038/s41598-023-36166-5

**Published:** 2023-06-07

**Authors:** B. L. Sánchez-Rodríguez, I. Castillo-Maldonado, D. Pedroza-Escobar, D. Delgadillo-Guzmán, M. F. Soto-Jiménez

**Affiliations:** 1 Centro de Investigacion Biomedica, Universidad Autonoma de Coahuila, Unidad Torreon, Torreon, Mexico; 2 Facultad de Medicina, Universidad Autonoma de Coahuila, Unidad Torreon, Torreón, Mexico; 3 Instituto de Ciencias del Mar y Limnología, Universidad Nacional Autónoma de Mexico, Av. Joel Montes Camarena, 82040 Mazatlán, Sinaloa Mexico

**Keywords:** Risk factors, Mass spectrometry, Natural hazards

## Abstract

Chronic endemic regional hydroarsenicism (CERHA) is a global issue that affects over 200 million people exposed to arsenic (As) in drinking water. This includes 1.75 million individuals residing in La Comarca Lagunera, a region in north-central Mexico. Arsenic levels in this region typically exceeds the WHO guideline of 10 µg L^−1^. Biochemical alterations related to the human As metabolism may increase the risk of overweight and obesity (O&O), type 2 diabetes (T2D), and hypertension (AHT). In our study, we investigated the role of As in drinking water as a risk factor for these metabolic diseases. We focused on populations with historically moderate (San Pedro) and low (Lerdo) drinking water As levels and people with no historical evidence of As water contamination. The exposure assessment to As was based on measurements of the drinking water (medians 67.2, 21.0, 4.3 µg L^−1^) and urinary As concentrations in women (9.4, 5.3, 0.8 µg L^−1^) and men (18.1, 4.8, 1.0 µg L^−1^). A significant correlation between As in drinking water and urine evidenced the As exposure in the population (R^2^ = 0.72). Adjusted odds ratios with 95% confidence intervals evidenced higher chances of being diagnosed with T2D (1.7, 1.2–2.0) and AHT (1.8, 1.7–1.9) in individuals living in San Pedro than those in Lerdo. Still, there was no significant association with obesity. Individuals living in CERHA towns were found to have a higher risk of obesity (1.3–1.9), T2D (1.5 to 3.3), and AHT (1.4 to 2.4) compared to those residing in non-CERHA towns. Finally, obesity is more probable in women [inverse of OR and 95%CI 0.4 (0.2–0.7)] compared to men, while men is more likely to be diagnosed with T2D [OR = 2.0 (1.4–2.3)] and AHT [OR = 2.0 (1.5–2.3)] than women, independently of the municipality.

## Introduction

The prevalence of overweight and obesity (O&O), type 2 diabetes (T2D), and hypertension (AHT) has significantly increased since the 1970s^1^, making it the leading health problem in Mexico, and still growing. The 2016–2018 National Health and Nutrition Surveys (ENSANUT)^1^ revealed that people with O&O accounted for 96 million (71.3 to 75.2%, or 3 out of 4 adults). Additionally, 13.5 million people (10.4%) were diagnosed with T2D, and 15.2 million people (12%) live with AHT. T2D is second leading cause of death in Mexico, with 106,525 fatalities reported in 2018^2^. Mexico has the sixth-highest global prevalence of T2D and the highest incidence of deaths among countries with large populations^3^.

The multifactorial determinants of O&O, T2D, and AHT include accelerated modification diet resulting from consumption of high-calorie, high-carbohydrate, and high-fat foods, sedentary lifestyle patterns, and genetic susceptibility, particularly among Amerindian-derived populations^3–5^. However, environmental factors may also influence genetic predispositions and contribute to the rapid increase in O&O, T2D, and AHT [e.g.,^6–9^].

Chronic endemic regional hydroarsenicism (CERHA) is related to the natural presence of arsenic (As) in groundwater for human consumption and is prevalent in many countries worldwide^10–13^. Over 200 million people are chronically exposed to As in drinking water at levels that exceed the World Health Organization guidelines of 10 µg L^−1^ for drinking water^10, 14, 15^. The population most severely affected by CERHA worldwide consist of low-socioeconomic status households. CERHA regions in the Americas include Argentina, Bolivia, Chile, El Salvador, the United States of America, Nicaragua, Peru, and Mexico. A CERHA region is located in central-north Mexico, specifically in the La Comarca Lagunera province. Nine municipalities within the Coahuila and Durango states, with a population of close to 1.75 million people^2^, have been affected by arsenic in groundwater for seven decades. Typical As concentrations in groundwater in La Comarca Lagunera province range from 0.7 to > 800 µg L^−1^ [e.g.,^16–20^]. Adverse health effects related to As exposure have been documented since the 1960s.

In this research, we investigated the probable association between As exposure in drinking water and the metabolic diseases of O&O, T2D, and AHT in the La Comarca Lagunera province. We recruited individuals living in municipalities within the CERHA region with a history of As exposure in drinking water, as well as those living in municipalities with no historical evidence of As water contamination. For this study, we selected the San Pedro municipality in Coahuila and Lerdo municipality in Durango, with historically moderate (San Pedro) and low (Lerdo) levels of As exposure in drinking water. Aditionally, four non-CERHA municipalities in La Comarca, with no historical evidence of As water contaminationwere included (Nazas, Cuencame, Simon Bolivar, and Mapimi). The selected municipalities have a population with relatively homogenous economical and sociocultural characteristics^1, 2, 21^.

The exposure assessment to As was based on measurements of drinking water and urinary As concentrations, analyzed using high-resolution inductively coupled plasma mass spectrometry. We hypothesized that individuals in San Pedro and Lerdo had higher rates of ingestion and urinary excretion of As than those in non-CERHA municipalities. In addition, we expected a higher prevalence of O&O, T2D, and AHT in populations exposed to higher As levels in San Pedro than in Lerdo, and both than in non-CERHA municipalities. Based on the ENSANUT 2018–2019 dataset^1^, we anticipated a higher prevalence of these pathologies in the CERHA region compared to non-CERHA municipalities in north-central Mexico.

To investigate the potential association between As exposure in drinking water and urine and O&O, T2D, and AHT, we conducted a logistic regression model (LRM) analysis. We recruited participants in the same age range and with relatively similar economic and sociocultural characteristics to control for significant individual-level confounding factors. LRM is a powerful tool in epidemiologic studies that involve two or more explanatory variables and the response variable, while also reducing the impact of confounding factors^22^.

## Methods

### Study area and CERHA region

**Figure 1:**
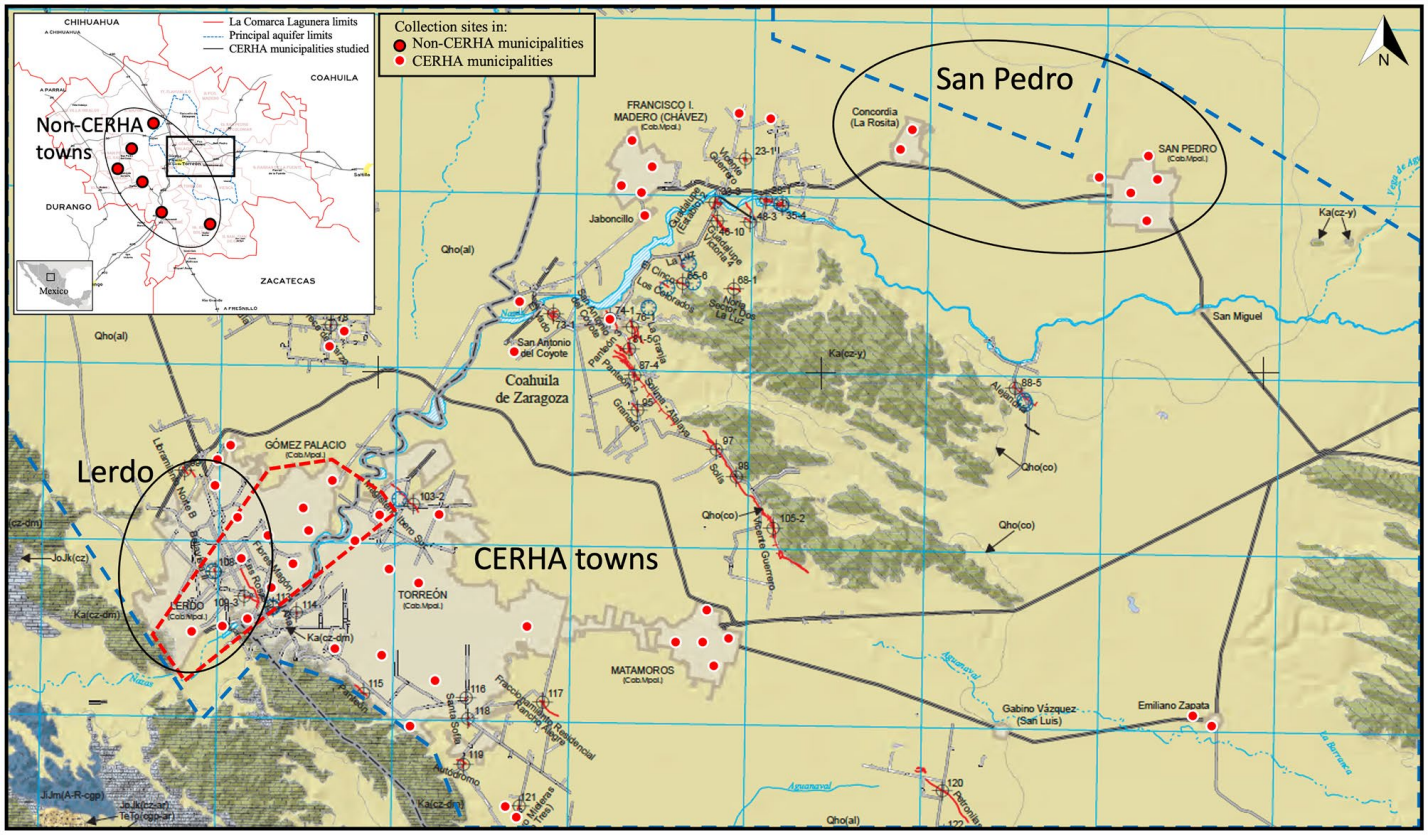
Location of the La Comarca Lagunera (103º 45′–102º W, 25º 15′–26º15′ N) between the northwest Durango and southwest Coahuila in the central-northern Mexico, Principal aquifer (polygon dotted in white), the main drinking water reservoir in the Comarca Lagunera province (polygon in blue), and Lerdo and San Pedro cities in Durango and Coahuila, respectively. The map has been modified from the Instituto Nacional de Estadística y Geografía (INEGI, 2018): “Mapa de susceptibilidad del fenómeno de subsidencia en el Valle de la Comarca Lagunera, Durango y Coahuila de Zaragoza (2018-12-19)”, which can be accessed at https://www.inegi.org.mx/app/biblioteca/ficha.html?upc=889463776345. The use of this map is in compliance with the "Terms of Free Use of INEGI Information".

La Comarca Lagunera province is in central-north Mexico, in the Chihuahua Desert (Fig. 1). The region is characterized by an arid to semiarid climate with scarce precipitation (250–500 mm y^−1^), high evaporation (> 1100 mm y^−1^), and average summer and winter temperatures of 31 and 16 ºC, respectively. Higher and lower precipitation occurs in July–August (13–52 mm/d, Julian days 190–220) and April (4 mm d^−1^, Julian 90–120). Before the Nazas and Aguanaval rivers were dammed, their flow discharges formed 13 ephemeral lagoons, including the Mayran lagoon, the largest in Latin America. These lagoons disappeared after the construction of the dams in the 1940s–1960s. In addition, the aquifer recharge in the region lowered rapidly after the 1960s. At the same time, the water demand tripled in the last 70 years due to the growth of agricultural and dairy cattle activities and the human population. Currently, water uses are agricultural-dairy cattle (91%) and urban and industrial activities (9%), with 60.6% of the volume extracted from the aquifers and 39.4% from the dams^23^.

In this study, we compiled As levels data in groundwater samples from wells in La Comarca Lagunera province from previous studies conducted by the Department of Biochemistry of the Universidad Autonoma de Coahuila Unidad Torreon and other researchers and government agencies. Our primary interest was to verify the historical exposure to As levels in the public water supplies in CERHA municipalities and distinguish those with no historical evidence of As water contamination in non-CERHA municipalities.

### Study population description

La Comarca Lagunera has 1,754,142 inhabitants and is comprised of five municipalities of Coahuila (60.7% of the population) and 15 municipalities of Durango (39.3%)^2^. About 70% of the population resides in Torreon (Coahuila) and Lerdo and Gomez-Palacio (Durango). The largest CERHA region in Mexico involves nine municipalities of the La Comarca Lagunera province, including Torreon, Matamoros, San Pedro de las Colonias, Francisco I. Madero, and Viesca in Coahuila and Gomez Palacio, Lerdo, Rodeo, and Tlahualilo in Durango (Fig. 1). Two CERHA municipalities were selected for this study, San Pedro (101,141 people) and Lerdo (163,313)^2^. In addition, non-CERHA municipalities in La Comarca Lagunera were selected to represent non-As exposure, including Nazas (12,894), Cuencame (34,955), Simon Bolivar (10,038), and Mapimi (26,932). The population is comprised of roughly equal numbers of men and women^2^.

San Pedro and Lerdo are municipalities with historically moderate and low water As levels. Nazas, Cuencame, Simon Bolivar, and Mapimi are municipalities with no historical evidence of As water contamination. Municipalities selection was primarily based on their As exposure background.

In addition, we accesed the dataset from the Report Annual of Multidimensional Poverty and Social Backwardness to obtain information on the selected municipalities regarding social deprivation indicators, housing quality and space, and conditions of multidimensional poverty^21^. The selected municipalities contain a population with relatively homogeneous economic and sociocultural characteristics (see Table S1). In terms of social deprivation indicators, between 13.9 and 22.6% of the population experiences educational backwardness, while only 16.7% to 32.2% and 10.9% to 29.3% have access to quality health services and nutritious food, respectively. In regards to basic housing services, the majority of people have access to water (between 84.3 and 91.1%), sewage drainage (between 86.5 and 98.0%), and electricity (99.8%). However, ensuring access to water remains a significant challenge for many municipalities in the region^20^. Based on multidimensional poverty measures, the majority of people experience either moderate poverty (between 33.2 and 45.6%) or are vulnerable due to a lack of basic services (between 31.7 and 45.6%). We accessed the database from the most recent ENSANUT 2018–2019 survey (https://ensanut.insp.mx/encuestas/ensanut2018/informes.php) to obtain the prevalence rates of O&O, T2D, and AHT in both CERHA and non-CERHA municipalities in La Comarca Lagunera, located in Coahuila and Durango states, as well as nationwide^1^.

### Recruitment and characteristics of participants

A flowchart detailing the enrollment and recruitment process for participants in our study is presented in Fig. S1. Public meetings were held in communities within the selected municipalities (e.g., in public squares and elementary schools) to explain the study objectives. Hundreds of individuals (n = 872) expressed interest in participating in this study. Each volunteer was informed of the study's objectives and procedures and signed a consent form. The procedures for refuse or exclusion from the study at any stage, even after the consent, were also explained to enrolled participants. Eligible participants (n = 724) were questioned about drinking water source (tap water or commercially purified water) and the daily ingestion rate. They completed the National Health and Nutrition Survey questionnaire^1^ to provide information on their access to nutritious food (item, frequency and portion), included dairy products (milk, cheese, yogurt), grains (corn, wheat, rice, beans) and all vegetables and fruits, beverages and snaks consumed per day, and fish and shellfish, meat, eggs and poultry consumed per week that may result in differences in the dietary intake of inorganic As (https://ensanut.insp.mx/encuestas/ensanut100k2018/descargas.php).

Participants also completed the survey of Multidimensional Poverty Thresholds of the National Council for the Evaluation of Social Development Policy (CONEVAL) (https://www.coneval.org.mx/rw/resource/coneval/med_pobreza/Cuestionario_Individual.pdf)^24^. This survey provide information on the job type (construction, informal sales, customer service, housewife) and work distance, social deprivation indicators (e.g., educational backwardness, access to health services, and social security), housing quality and space (e.g., dirt floors, non-durable roof and wall materials, overcrowded homes, and basic services such as water, sewage drainage, and electricity), and conditions of multidimensional poverty (e.g., extreme, moderate, vulnerable by income, or due to lack of basic services, not poor, and not vulnerable). Participants were also asked about their alcohol consumption and cigarette smoking habits (never, past or current use, duration of use, and cigarettes per day). Additionally, physical activity information was obtained for each individual in accordance with the Global Physical Activity Questionnaire (GPAQ) (https://www.who.int/publications/m/item/global-physical-activity-questionnaire)^25^.

The initial eligibility criteria for potential participants in this cohort study were: (1) individuals between the ages of 19 and 74 years old, (2) residents of the study area for at least five years, (3) individuals with relatively homogeneous economic and sociocultural characteristics, and (4) those who where not pregnant and had no critical health conditions (Fig. S1). Limitations of our study included a relatively small sample size across different age ranges. For instance, we faced recruitment challenges among individuals younger than 40, especially men, as they often study or work away from their hometowns, resulting in different As exposures. To ensure reliable statistical analysis and control for age-related confounding factors that might affect study outcomes, we selected a subset of participants aged 45–64 years (n = 287). Thus, excluded persons (n = 437) included younger than 45 and older than 64 years (358), pregnant women (2), extremely poor or non-poor people (17), alcoholics (18) and chronic smokers (30), diagnosed with chronic or acute diseases such as cancer and kidney disease (stones, infection, or failure) or amputated (10). The surveys also included questions regarding occupational use of agrochemicals and other toxic substances (current use status, duration of use, and specific chemicals). People working regularly with agrochemicals and other toxic substances were also excluded (2).

### Physical assessment and anthropometric measures

The recruited individuals were physically screened by medical practitioners from the Faculty of Medicine at the Universidad Autonoma de Coahuila. They were examined for anthropometric measures. Bodyweight was measured using an electronic digital scale, and standing height was measured using a stadiometer. Overweight and obese were assessed using the body mass index (BMI), calculated by dividing the weight (kg) by the square of the height in meters (kg/m^2^). BMI is a measure to indicate nutritional status in adults. A person is considered underweight when their BMI is < 18.5, normal weight is 18.5–24.9, overweight or pre-obese is 25.0–29.9, obesity class I is 30.0–34.9, obesity class II is 35.0–39.9, and obesity class III is ≥ 40^26^.

Systolic (SBP) and diastolic (DBP) blood pressure were measured during morning before eating or taking any medications. SBP and DBP were measured using standardized techniques and equipment, specifically the Omron HEM907 XL digital sphygmomanometer. Recorded values were an average of two readings taken with a 5-min interval between each. According to the American Heart Association, BP levels in mm Hg are normal for systolic when < 120 and diastolic when < 80, elevated when 120–129 and < 80, and high blood pressure (hypertension) when ≥ 130 and ≥ 80, respectively^27^. The total prevalence of AHT was obtained by adding the prevalence of diagnosed AHT, as declared in the questionaries, plus the prevalence of undiagnosed AHT in participants who answered “NO” to diagnosed AHT but had an SBP ≥ 130 and DBP ≥ 80 mm Hg. The time of diagnosis for metabolic diseases was also inquired about.

### Collection and analysis of biological samples

The participants were asked to provide fasting blood samples. A venipuncture (2 mL) blood sample was collected from each participant for fasting serum blood glucose (FSBG) analysis. FSBG was measured by using an enzymatic-colorimetric method (glucose kit Spinreact and SPIN640Plus Autoanalyzer). A blood sugar level < 126 mg dL^−1^ is normal, FSBG (8–12 h) ≥ 126 to 199 mg dL^−1^ indicates prediabetes, and > 200 mg dL^−1^ indicates T2D. We defined T2D prevalence as diagnosed T2D when T2D diagnosis was self-reported in the questionnaires and undiagnosed T2D for participants who answered “NO” in the self-reported questionnaire, but had an FSBG result ≥ 126 mg dL^−1^.

Participants were also asked to provide the urine excreted in a day. Urine samples were immediately stored at 4ºC in portable coolers in the field. Creatinine in urine 24-h samples was measured using a Roche/Hitachi Modular P Chemistry Analyzer using an enzymatic (creatininase) reaction. At the Biomedical Investigations Center (CIB lab of the University Autonomous of Coahuila), urine aliquots were transferred to 50 ml acid-washed tubes and frozen at − 20 °C until arsenic analysis. All urine samples were kept in a freezer until their shipment to the Stables Isotope Laboratory in Mazatlan of the ICMyL-UNAM.

Individuals who did not provide biological samples during one of the three sampling surveys during 2015 to 2017 were removed from the study. During the study, 30 participants were excluded because they did not provide biological samples during one of the three sampling surveys.

### Collection of groundwater and drinking water in the CERHA region

Between 2005–2007 and 2015–2017, representative groundwater and drinking water samples (1 L LDPE Nalgene bottle) were collected from wells located in CERHA and non-CERHA municipalities, including Lerdo, Gomez Palacio, Nazas, Cuencame, Simon Bolivar, and Mapimi in Durango and San Pedro, Torreon, and Viesca in Coahuila state. Drinking water samples were collected from each participant's home in San Pedro (27 towns), Lerdo (4 towns), and non-CERHA (12 towns) municipalities. Samples were double-bagged with clean plastic zip-lock bags, labeled (site, GPS position, date, and hour), and kept at 4ºC in hermetically box ice. Water samples were transported to the Stables Isotope Laboratory in Mazatlan of the ICMyL-UNAM.

### Analysis of As in drinking water and human urine

Drinking water and human urine samples were processed and analyzed in HEPA-filtered air (Class1000) and trace metal clean laboratories^28^. Drinking water samples were filtered through a 0.45 μm filter and acidified to pH ~ 2 with 1 mL nitric acid (HNO_3_ 67–70%, Optima™, for Ultra Trace Elemental Analysis, Fisher Chemical™) per 1 L of water, then stored in acid-cleaned HDPE bottles within 24 h after sampling. Urine samples were stored and kept frozen until analyzed. Aliquots of urine samples (5 mL) were digested with 10 mL concentrated HNO_3_ in a block digester at 120 ºC for 4 h. The digested samples were evaporated to dryness and then dissolved with 1M HNO_3_ for As concentration measurements. High-purity reagents (Ultra Trace Elemental Analysis) and water (resistivity ≥ 18 MΩ cm^−1^ at 25 °C, Academic Milli-Q, Millipore, Bedford, MA, USA) were used. The analytical determinations of As in digested urine and filtered and acidified drinking water were conducted using a High-Resolution Inductively Coupled Plasma Mass Spectrometry (HR-ICP-MS, Thermo Element XR, Bremen, Germany)^28^. The instrument was operated in a high-resolution mode (R = 10,000). Quantification was performed using an external calibration curve (0.5, 1, 2, 5, 10, and 25 µg As L^−1^) obtained from 1000 mg L^−1^ single patron solution (High Purity Standards, Charleston, SC, USA) and acidified with 1% HNO_3_. Before analysis, the standard solutions and sample were spiked with ^115^In as the internal standard (final concentration of 1 µg L^−1^) to correct for instrumental drifts. Field and laboratory blanks and two certified reference materials (NIST-1640a Trace Elements in Natural Waters and NIST-2668 Toxic Elements in Frozen Human Urine) were also measured with our studied samples. Arsenic recovery was > 95%, and the coefficient of variation was < 10% in both CRMs. The method detection limits were < 10 ng L^−1^ for As. We normalized As levels in urine by creatinine concentration (units in µg of As per gram of creatinine).

### Calculations of As ingestion and cumulative exposure

To estimate the average daily ingestion of As (ADI, µg As d^−1^ kg^−1^) from drinking water, we multiplied the daily drinking water ingestion rate (L d^−1^) by the concentration of As in the drinking water (µg L^−1^) and then divided the result by the individual’s body weight (kg). This calculation was performed for individuals in different groups based on their sex, location, and health status in terms of metabolic diseases. We then calculated the cumulative exposure to As (mg As kg^−1^) in a time-weighted manner by multiplying the ADI by the duration of tap or purified water consumption and dividing it by the residence time. The cumulative exposure to As was used as a predictor factor in the development of metabolic diseases with a long latency time, such as O&O, T2D and HTA.

### Statistical analysis

The final recruited participants (n = 257) were grouped according to the As exposition level in drinking water (San Pedro: moderate, Lerdo: low, and no-CERHA municipality: non-exposed), diagnoses or absence of O&O, T2D, and AHT, and sex (women or men). Descriptive statistics were performed among groups to assess the demographic, socioeconomic status, nutrition and lifestyle. In addition, a statistical analysis of the anthropometric measures and clinical analysis (e.g., BMI, SBP, DBP, FSBG, and creatinine) As levels in drinking water and urine, and the ADI and cumulative exposure to As were conducted for the different groups. We evaluated differences between groups based on the level of As exposure in drinking water (moderate, low, and non-exposed), as well as the presence or absence of metabolic diseases, while accounting for gender.

To provide a regional context for the exposure to As in tap water (groundwater) and commercially purified water (bottled) collected in the same and different locations, a multiple comparison was also performed. Because the assumptions of normality and homogeneity of variance were not met and the number of participants in each group was different, we used the non-parametric Kruskal–Wallis test to evaluate the differences between groups. If the Kruskal–Wallis test detected a significant difference, we used Dunn's post-hoc test to adjust the *p*-values for multiple comparisons.

Simple linear regression analyses were performed to establish the relationships between the As uptake (ADI and cumulative exposure doses) via drinking water and its urinary excretion before and after normalization for creatinine.

Spearman's rank correlation analysis was also employed to evaluate the bivariate associations between As levels in urine, cumulative exposure doses to As and creatinine with all potential predictors, including anthropometric measures (weight, height, BMI) and clinical analysis (creatinine, glucose concentrations, blood pressure), diet (e.g., grains, vegetables, fruits, dairy products, meat, poultry, seafood), drinking tap water or purified water (no, yes) and ingestion water rate, demographic (age, gender) and socioeconomic (e.g., education, poverty level), job type (e.g., unemployed, housekeeping, field worker, informal sales, industrial or maquila worker) and work distance (close, faraway), and lifestyle (e.g., drinking alcohol, smoking, excersise).

We used logistic regression model (LMR) analysis was used to examine the association between each metabolic disease (O&O, T2D, and AHT) and the As exposure in drinking water and As in urine (predictor variables), adjusted to sex, location, and residence time^24^. Factors that can be associated with metabolic disease were also separately adjusted, including diet, lifestyle, socio-demographic and economic status. Thus, most significant individual-level confounding that could explain variations in metabolic diseases were controlled in this study.

The arsenic exposure entered the model either as a continuous (As levels in drinking water), categorical (moderate, low, and non-As levels in drinking water), or dichotomous (presence/absence) exposure variable. For the categorical exposure variables, we assumed moderate (> 25 to 125 µg L^−1^), low (> 10–25 µg L^−1^), and below the WHO guideline value of 10 µg L^−1^.

We obtained the odds ratio (OR) and its corresponding 95% confidence interval (95% CI) by LRM analysis. For a binary response variable, such as a response to a yes–no question to the prevalence of O&O, T2D, and AHT, the logistic regression model is as follows:

1 $$log\frac{Prob(Y=r1)}{Prob(Y=r2)}=Xb$$

where $r$1 and $r$2 are the two response levels. Then, the odds are calculated as follows:

2 $$\frac{{Prob\left( {Y = r1} \right)}}{{Prob\left( {Y = r2} \right)}} = \exp \left( X\beta \right) = \exp \left( \beta_0 \right) \cdot \exp \left( {\beta_1X_1} \right) \dots \exp \left( {{\beta_\text{i}}X_{\text{i}}} \right)$$

Note that exp(*βi*(*Xi* + 1)) = exp(*βiXi*) $\cdot$ exp(*βi*). The odds multiplied by exp(*βi*) is the unit odds ratio, and multiplied by exp((*X*_high–_*X*_low_)*βi*), is the range odds ratio. The OR magnitude is commonly referred to as the “strength of the association.” An OR ~ 1 indicates that there is no association between exposure to As and the disease, while OR > 1 indicates that exposure may be a risk factor for the disease. Conversely, an OR < 1 implies exposure may be a protective factor against the disease. Wald X^2^ test was employed to assess the significance of each variable. The Wald test is a significance test for individual regression coefficients in LRM. All statistical analyses were performed using JMP version 14 software (SAS Institute, Cary, NC, USA) with *p* values < 0.05 considered statistically significant.

### Bioethical aspects

Based on the Declaration of Helsinki: ethical principles for medical research involving human subjects^29^. The experimental protocol was approved by the Ethical Committee of the Autonomous University of Coahuila.

## Results

### General characteristics of participants

Table S2 shows the characteristics of the recruited participants in this study, stratified by location and gender. Based on the As levels in drinking water, the pool of subjects was categorized as 30% with moderate exposure, 43% with low exposure, and 27% without exposure to As from drinking water. The recruited participants consisted of 50–60% women and 40–50% men, ranging in age from 45 to 64 years old and with a residence time ranging from 38 to 43 years, indicating that at least half of their lives were spent in the studied towns. Adults between the ages of 45 and 64 represent one of the most significant demographic strata in Mexico (20% of total adults with 11.63 and 13.15 million of men and women, respectively), the mature working age, and more vulnerable group with T2D, as the first leading cause of death^2^.

Based on surveys completed by recruited participants, the socioeconomic characteristics of most participants were representative of the general population (Tables S1 and S2). Regarding social deprivation indicators, < 10% had educational backwardness (< 6 y), over 70% had completed 8–12 y, and < 20% had more than 12 y of education. Between a third to half of the participants had limited access to health services and social security. All houses of participants had access to basic housing services such as water, sewage drainage, and electricity, and their homes were constructed with durable materials. Participants were distributed relatively evenly between moderate poverty and vulnerability due to a lack of basic services. The job type in participants both sexes were 35–40% unemployed, women: 30–40% housekeeping, < 10% field worker, < 10% informal sales, men: < 30% industrial or maquila worker, and < 10% field worker, < 10% informal sales).

Similarities were observed in the diets of the participants with respect to the types of food consumed and ingestion rates (Table S3). The diets were primarily based on corn, rice, beans, meat, and poultry, with a restricted variety of vegetables and fruits. Consumption of fish and shellfish was practically non-existent. Considering the frequency and portion size of each food item, about 30% of the participants has access to nutritious and quality food (28.1–33.0%). A study limitation is that all dietary items were self-reported with a degree of imprecision.

Based on the completed study questionnaires, the source of drinking water and daily ingestion rate (L d^−1^) were obtained in the recruited participants (Table S4). In San Pedro, 55–70% of the participant’s drink purified water (bottled). In Lerdo and non-CERHA municipalities, ≥ 65% of the recruited participants drinks tap water. Most people declaring drinking purified waters also recognized the use of tap water for food preparation (e.g., rinsing and cooking food and dishwashing). The daily water intake in participants averaged 1.2–1.8 and 1.6–2.2 L d^−1^ for women and men, respectively. Men drink significantly more water than women (*p < *0.05) and but non-significant differences were observed between municipalities (*p >* 0.05).

The results of alcohol drinking and smoking habits showed that 10–12% of the participants consumed alcohol (1–5 times per month for 17–19 years) and 8–10% tobacco (1–5 cigarettes per day for over 15 y), with no significant differences between municipalities (Table S5).

Most people practice less physical activity than the WHO recommendation^25^ for adults aged 18–64, which is at least 150 min of moderate-intensity aerobic physical activity throughout the week or at least 75 min of vigorous-intensity aerobic physical activity throughout the week (Table S6). Only 16% of the participants reported engaging in regular exercise, with 10–19.5% of women and 12.5–18.2% of men participating in moderate- to vigorous-intensity activities. The remaining 84% reported low intensity physical activity or a sedentary lifestyle. No significant differences were observed between sexes and municipalities (*p >* 0.05).

### Arsenic levels in groundwater and drinking water

A statistical summary of the As levels in groundwater and drinking water samples, collected and analyzed in this study and compiled data from previous studies, are shown in Table 1. Based on multiple (pairwise) comparisons using Kruskal–Wallis test, groundwater samples collected in San Pedro (medians 67.2–172.9 µg L^−1^) and Lerdo (19.0–21.0 µg L^−1^), were comparable between surveys for the same municipality (*p >* 0.05). However, As levels in San Pedro were three times higher than those in Lerdo municipality (*p < *0.05). In Nazas, Cuencame, Simon Bolivar, and Mapimi, the As levels in groundwater (2.0–10.7 µg L^−1^) were significantly lower than in San Pedro and Lerdo municipalities (*p < *0.05). Regarding As levels in drinking water, values in San Pedro (30.0–42.2 µg L^−1^) were two times higher than those in Lerdo (16.8–19.4 µg L^−1^). Arsenic levels in tap water in non-CERHA municipalities (1.2–10.0 µg L^−1^), were significantly lower than in Lerdo and San Pedro (*p < *0.05). No significant differences were observed in the median As levels in groundwater collected from CERHA and non-CERHA municipalities in the La Comarca region during our 2005–2007 and 2015–2017 surveys, as well as in the compilated historical dataset (*p >* 0.05).

**Table 1:** Median (10th–90th) values of As levels in groundwater (tap water) and commercially purified water (bottled water) samples collected in 2005–2007 and 2015–2017 surveys, along with existing historical dataset (1960s–2000) samples from wells located in municipalities of the La Comarca Lagunera province (north Mexico). Units µg L^−1^.

Drinking water source		N	Historic dataset	N	2005–2007 survey	N	2015–2017 survey
Durango							
Gomez Palacio	Tap water	250	33.1 (1–532)^c^	5	15.3 (8.2–36.8)^b^	7	31.9 (7.9–101.3)^c^
Lerdo city	Tap water	189	34.3 (1–400)^c^	18	19.0 (1.0–47)^b^	19	21.0 (0.9–51.6)^b^
	Bottled water		Non-available data	30	19.4 (7.1–29.5)^b^	30	16.8 (0.5–40.0)^b^
Tlahualilo	Tap water	199	131.6 (1–770)^d,e^	3	24.4 (12.5–35)^b,c^	8	81.1 ± 27.7 (35.1–123.0)^d^
Francisco I Madero	Tap water	331	126 (1–880)^d,e^	26	40.6 (1.0–206.0)^c^	31	83.0 (8.2–218.3)^c,d^
*Nazas	Tap water	31	4.3 (nd to 15)^a^	4	7.5 (2–15.1)	6	8.4 (6.5–10.2)^a^
*Cuencame	Tap water	21	8.5 (nd to 47.1)^a,b^		No samples collected	6	10.7 (1.3–17.7)^a,b^
*Simon Bolivar	Tap water	16	< 5 (nd to 5.0)		No samples collected	6	2.7 (0.1–7.7)^a^
*Mapimi	Tap water	21	4.6 (nd to 10.0)^a^		No samples collected	6	2.0 (0.05–9.2)^a^
Non-CERHA municipalities	Bottled water		Non-available data		No samples collected	14	7.0 (1.2–10.0)^a^
Coahuila							
Matamoros	Tap water	168	62.9 (1.0–465.0)^d^	6	29.9 (7.8–38.7)^c^	13	38.0 (8.6–101.2)^c^
San Pedro	Tap water	324	117.2 (0.6–800.0)^d,e^	17	172.9 (7.1–392.0)^e^	23	67.2 (27.9–101.1)^d^
	Bottled water		Non-available data	11	42.2 (17.9–181.0)^c^	60	39 (2–94.6)^c^
Torreon	Tap water	504	16.8 (0.3–258)^b^	98	16.7 (3.1–52)^b^	12	35.8 (19.7–51.9)^c^
Viesca	Tap water	79	67.2 (1.0–450.0)^d^		No samples collected	4	62.5 (44.3–111.4)^d^
Non-CERHA municipalities			Non-available data	14	3.5 (0.1–8.1)^a^	11	4.3 (1.2–8.2)^a^

Multiple comparisons performed by Kruskal–Wallis test confirmed by Dunn's post-hoc test. Significant differences (*p < *0.05): a < b < c < d. *Non-CERHA municipalities. nd: non-detectable.

### Arsenic uptake and urinary excretion

Using the volume of drinking water and their corresponding As levels, years of residence, and body weight, the average daily ingestion of As and the cumulative exposure doses per person were calculated for individuals of the different groups. The medians of the groups diagnosed with and without O&O, T2D, and HTA across three independent localities, we compared by Kruskal–Wallis and Dunn's post-hoc test to adjust the *p*-values for multiple comparisons, while also taking gender into consideration (Table 2). In San Pedro with moderate exposure to As in drinking water, the ADI and cumulative doses of As were significantly higher than low exposed people in Lerdo, and both were higher than people in non-CERHA municipalities. ADI values and cumulative doses in San Pedro (medians 0.54–0.59 µg kg^−1^ d^−1^ and 7.2–7.4 mg kg^−1^) were two times than in Lerdo (0.22–0.26 and 2.9–4.0) and close to 10 times than in non-CERHA municipalities (0.05 and 0.6, respectively). Non-significant differences were observed between the sexes in any location.

**Table 2:** Median (10th-90th) values of average daily ingestion of arsenic (µg As kg^−1^ of body weight d^−1^), time-weighted cumulative exposure doses to As (mg As kg^−1^ of body weight) during the drinking tap or purified water consumption, As in urine (µg U-As L^−1^), creatinine level (g L^−1^), and As in urine normalized to creatinine (µg U-As g^−1^ U-creat) for women and men from San Pedro (moderate As exposure), Lerdo (low As exposure) and non-CERHA municipalities (Nazas, Cuencame, Simon Bolivar, and Mapimi).

	Women	Men
Arsenic average daily ingestion (µg kg^−1^ d^−1^)		
San Pedro, Coahuila	0.54(0.06–2.86)^c^	0.59(0.04–2.5)^c^
Lerdo, Durango	0.22(0.04–0.64)^b^	0.26(0.05–0.56)^b^
Non-CERHA municipalities	0.05(0.01–0.12)^a^	0.05(0.01–0.14)^a^
Cumulative exposure doses of As (mg kg^−1^ d^−1^)		
San Pedro, Coahuila	7.2(0.6–35.7)^c^	7.4(0.5–39.9)^c^
Lerdo, Durango	2.9(0.4–10.0)^b^	4.0(0.3–11.1)^b^
Non-CERHA municipalities	0.6(0.0.1–1.4)^a^	0.6(0.2–2.1)^a^
As in urine (µg U-As L^−1^)		
San Pedro, Coahuila	9.4(2.3–55.7)^b^	18.1(2.2–43.5)^b^
Lerdo, Durango	5.3(2.3–14.2)^a^	4.8(1.9–12.1)^a^
Non-CERHA municipalities	0.8(0.1–2.3)^a^	1.0(0.3–3.1)^a^
Creatinine levels (g L^−1^)		
San Pedro, Coahuila	0.54(0.21–0.93)^b^	0.8(0.38–1.03)^b^
Lerdo, Durango	0.6(0.28–0.89)^b^	0.67(0.21–0.94)^b^
Non-CERHA municipalities	0.29(0.12–0.68)^a^	0.74(0.26–0.92)^b^
As in urine normalized to creatinine (µg U-As g^−1^ U-creat)		
San Pedro, Coahuila	13.0(7.8–116)^b^	15.9(5.0–49.3)^a,b^
Lerdo, Durango	11.2(8.0–95.7)^a,b^	10.9(2.2–34.3)^b^
Non-CERHA municipalities	8.0(6.6–27.5)^b^	6.8(1.5–8.9)^b^

Multiple comparisons performed by Kruskal–Wallis test confirmed by Dunn's post-hoc test. Significant differences (*p < *0.05): a < b < c.

The urinary As excretion in San Pedro people (median 9.4–18.1 µg U-As L^−1^) was 2–3 times higher than those in Lerdo (4.8–5.3 µg U-As L^−1^) people and > 10 times (0.8–1.0 µg U-As L^−1^) than in non-exposed people (Table 2). Non-significant differences were observed between the sexes. Comparatively, the medians of the urine creatinine levels showed not significant differences between localities exposed and non-exposed to As in drinking water and sex (medians 0.6 to 0.8 g L^−1^), except in non-exposed women with minimum of 0.29(0.12–0.68)   g  L^−1^ (Table 2). Levels of As in urine normalized to creatinine (µg U-As g^−1^ U-creat), also included in Table 2, showed a large variability with medians from 6.8 to 15.9 µg U-As g^−1^ U-creat. Non-significant differences were observed in the normalized As in urine exposure and non-exposure or between the sexes (*p >* 0.05).

**Figure 2:**
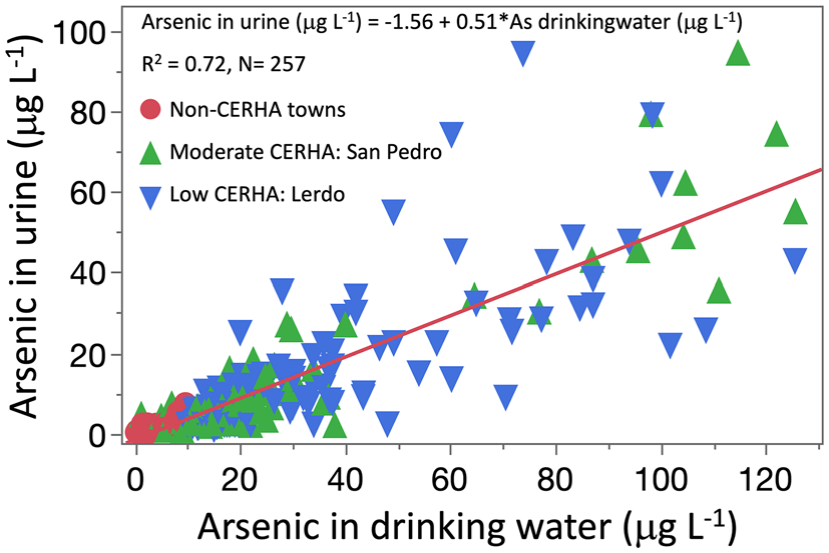
Analysis of regression linear between arsenic in urine (µg U-As L^−1^) and drinking water (µg L^−1^).

Arsenic levels in drinking waters and the uptake As rates were reflected in the urinary As excretion. For example, a linear regression analysis between As in urine and drinking water [U-As (µg  L^−1^) = − 1.56 + 0.51*As drinking water (µg  L^−1^), R^2^ = 0.72, N = 257] reflected a significant association (Fig. 2). Levels of urinary As normalized by urine creatinine concentration [µg of As g^−1^ creatinine in urine) in function of As in drinking water, showed also a significant association [U-As normalized to creatinine (µg U-As g^−1^ U-creat) = 5.46 + 0.32*As drinking water (µg L^−1^), R^2^ = 0.68, N = 257], but lower than the non-normalized levels of As in urine.

The association between urinary As levels, cumulative exposure to As, and creatinine with all potential predictors was evaluated using Spearman’s rank correlation analysis. The significant Spearman’s correlation coeficients (ρ values) are listed in Table 3. Creatinine was found to be significantly associated with urinary As (ρ = 0.51, *p* = 0.0044), which invalidates the normalization of urinary As with creatinine. Furthermore, As in urine was positively correlated with the cumulative doses to As (ρ = 0.67, *p < *0.0001). Cumulative doses to As were found to be positively correlated with As in urine and in drinking water, the ingestion rate of tap water, creatinine, As in urine normalized to creatinine, residence time, body weight (0.20 < ρ < 0.85, *p < *0.0014). Aditionally, the cumulative doses to As were negatively correlated with volume and time of consumption of bottled water. Creatinine was found to be positively correlated with As in drinking water, individual body weight and intake of all fruits and grains and alcohol (0.37 ≤ ρ ≤ 0.69, *p* ≤ 0.0347). Finaly, As in urine was positively correlated with the time of diagnosis of T2D (ρ = 0.56, *p* = 0.0015).

**Table 3:** Association between urinary As levels, cumulative exposure to As, and creatinine (predictor variables) with potential predictors. Only significant Spearman correlations (ρ) were enlisted.

Variable	vs. variable	ρ Spearman	Prob >|ρ|
As in urine (µg L^−1^)	As in drinking water (µg L^−1^)	0.84	0.0001
	Diagnosed T2D time (y)	0.56	0.0015
	Creatinine (g L^−1^)	0.51	0.0044
	Cumulative doses to As (mg kg^−1^ d^−1^)	0.67	< 0.0001
Cumulative doses to As (mg kg^−1^ d^−1^)	As in drinking water (µg L^−1^)	0.85	< 0.0001
	Drinking tap water (no, yes)	0.40	< 0.0001
	Ingestion water rate (L d^−1^)	0.30	< 0.0001
	Drinking purified water (no, yes)	-0.20	0.0014
	Time drinking purified water (y)	-0.20	0.0012
	Creatinine (g L^−1^)	0.37	0.0001
	As in urine normalized to creatinine (µg g^−1^)	0.66	0.0001
	Residence time	0.23	0.0014
	Body weight (kg)	0.20	0.0057
Creatinine (g L^−1^)	As in drinking water (µg L^−1^)	0.51	0.0041
	Weight (kg)	0.39	0.0347
	All fruits intake (portion d^−1^)	0.47	0.0092
	All grains intake (portion d^−1^)	0.37	0.0420
	Alcohol intake (event week^−1^)	0.69	0.0288

### Prevalences of O&O, T2D, and AHT

Multiple comparisons of medians of As in urine among population groups with different pathologies, performed by Kruskal–Wallis test and differences confirmed by Dunn's post-hoc test (*p < *0.05), are shown in Table 4. In San Pedro, non-obese women excreted more As in urine than obese women. However, non-significant differences were observed between obese and non-obese men (*p >* 0.05). Diagnosed T2D adults showed higher urinary As levels than non-diagnosed T2D (*p < *0.05). High variability in the urinary As levels were observed between diagnosed and undiagnosed AHT adults without significant differences. In Lerdo, non-significant differences were observed between sex for the same pathology or among pathologies for the same sex (*p >* 0.05). The urinary As excretion in non-exposed people neither showed significant differences between sex and pathologies. In all groups in San Pedro, the levels of urinary As were significantly higher than in Lerdo people (*p < *0.05). Likewise, all the urinary As excretion values in non-exposed people were significantly lower than people with moderate and low exposure to As in drinking water in San Pedro (7–13 times higher) and Lerdo (2–5 times higher) (*p < *0.05), respectively.

**Table 4:** Median (10th–90th) levels of arsenic in urine in moderate exposure people from San Pedro, Coahuila, and low exposure people from Lerdo, Durango, diagnosed or not with obesity, T2D, and AHT.

Sex	Pathology	San Pedro city	Lerdo city
		Median (10th–90th)	Median (10th–90th)
Female	Undiagnosed obesity	24.5 (2.0–94.6)^d^	8.0 (2.3–27)^b^
	Diagnosed obesity	13.6 (2.3–48.8)^c^	5.3 (0.5–15.9)^a^
Male	Undiagnosed obesity	19.2 (2.4–42.7)^c,d^	6.0 (1.7–18.2)^a,b^
	Diagnosed obesity	19.7 (4.1–47.8)^c,d^	5.0 (2.9–7)^a^
Female	Non-T2D	14.5 (2–62.1)^c^	6.5 (0.5–27)^a,b^
	Diagnosed T2D	25.0 (2.3–94.6)^d^	5.7 (0.7–8.3)^a,b^
Male	Non-T2D	16.0 (2.4–29.2)^c,d^	6.4 (1.9–18.2)^a,b^
	Diagnosed T2D	22.1 (4.1–47.8)^d^	5.0 (1.7–7-0)^a^
Female	Undiagnosed AHT	20.7 (2–79.3)^c,d^	6.8 (0.5–27)^a,b^
	Diagnosed AHT	15.5 (2.3–94.6)^c^	4.5 (3–6.3)^a^
Male	Undiagnosed AHT	13.5 (2.4–25.5)^c^	9.9 (5.1–18.2)^b^
	Diagnosed AHT	26.0 (4.1–47.8)^d^	3.3 (1.7–7)^a^

Multiple comparisons performed by Kruskal–Wallis test confirmed by Dunn's post-hoc test. Significant differences (*p < *0.05): a < b < c < d. Non-significant differences between sex and pathologies were observed in the urinary As excretion in non-exposed people. Median (10th-90th) in non-CERHA municipalities (Nazas, Cuencame, Simon Bolivar, and Mapimi): 1.9 (0.5–2.3) µg L^−1^.

The total prevalence of O&O, T2D, and AHT in San Pedro, Lerdo and non-CERHA municipaties in La Comarca province are shown in Table 5. According to the BMI classification, only 8.6% of women and up to 38.5% of men showed normal weight, 34 and 38.5% were overweight, and 57.4 and 23% were obese, respectively. In Lerdo, 8.7% of women and 25% of men showed normal weight, while 31.8 and 50% were overweight, and 59.5 and 25% showed obesity, respectively. In non-CERHA municipaties, 10.3% of woman and 31% of men showed normal weigth, but 40.7 and 43.4% showed overweight, and 49 and 25.6% were obese, respectively. Non-significant differences was observed in in the obesity prevalence in San Pedro and Lerdo people (*p >* 0.05), however, both were higher than people in non-CERHA municipalities in La Comarca (*p < *0.05).

**Table 5:** Total prevalence (% of the recruited participants) of overweight, obesity (total and classes), type 2 diabetes (T2D), and arterial hypertension (AHT) in women and men inhabiting San Pedro (moderate exposure to As in drinking water) Lerdo (low exposure) and non-CERHA (non-exposure) municipalities.

Health condition	San Pedro		Lerdo		Non-CERHA	
	Women	Male	Women	Male	Women	Male
Normal weight	8.6	38.5	8.7	25	10.3	31
Overweight	34	38.5	31.8	50	40.7	43.4
Obesity	57.4	23	59.5	25	49	25.6
class I	29.8	12.4	40.9	12.5	33.7	12.8
class II	17	10.6	18.6	12.5	15.3	11.6
class III	10.6	0	0	0	0	1.2
T2D	18.3	33.6	13.6	20.3	11.1	15.3
AHT	30.8	40.2	18.2	33.5	17.2	28.3

The combined prevalence of O&O is 76.5% (women 91.4%, men 61.5%), 83.1% of the adult population (women 91.3%, men 75%), and 79.4% of the adult population (women 89.7%, men 69%) in San Pedro, Lerdo, and non-CERHA municipalities, respectively.

Based on the applied questionaries, the prevalence of diagnosed T2D accounted for 27.3 (women 18.3 and men 33.6%) in San Pedro, 18.4% (women 13.6 and men 20.3%) in Lerdo, and 11.7% (women 11.1 and 15.3 men) in non-CERHA municipalities. At least 5% of the population answered “NO” for T2D diagnosis in the questionaries, resulting in pre-diabetic or diabetic based on FSBG levels (> 126 mg dL^−1^). In addition, 13–17% of participants showed values that indicate uncontrolled diabetes (FSBG > 200 mg dL^−1^). The declared AHT prevalence accounted for 34.9% in San Pedro (women 30.8% and men 40.2%), 29.9% in Lerdo city (women 18.2 and men 33.5%), and 21.7% (women 17.2 and 28.3% men) in non-CERHA municipalities. Based on measurements of SBP and DBP, 2–3% of participants declaring “NO” to AHT questions were added to the AHT percentages in both cities. Although probable errors in the diagnostic could occur in this study (non-diagnosed or misdiagnosed), the prevalence of T2D and AHT in adults increased slightly regarding the questionary declarations. A higher prevalence of T2D and AHT was observed in San Pedro concerning Lerdo municipality. Besides, non-exposed people showed significantly lower incidence of T2D and AHT than exposed people to moderate and low As levels in drinking water in San Pedro and Lerdo, respectively.

A statistical summary of the ENSANUT 2018–2019 survey, in terms of prevalences of O&O, T2D, and AHT, was performed for CERHA (moderate and low exposure to As in drinking waters) and non-CERHA municipalities in La Comarca province, Durango and Coahuila states, and Mexico (Table 6). Non-significant differences were observed in the prevalence of O&O, T2D, and AHT in San Pedro and Lerdo regarding other CERHA municipalities with moderate and low As exposure, respectively. However, the prevalence of those metabolic diseases was significantly higher in CERHA municipalities than in non-CERHA municipalities in La Comarca province, Durango and Coahuila states, and nationwide.

**Table 6:** Statistical summary of the ENSANUT 2018–2019 survey dataset of the total prevalence (% of total population ≥ 20 years) with diagnostic of obesity, type 2 diabetes (T2D), and arterial hypertension (AHT) in Mexico, Durango and Coahuila states, Lerdo and San Pedro municipalities and in CERHA and non-CERHA regions in both states. Mean ± SE (lower to upper confidence intervals).

	Obesity (%)	T2D (%)	AHT (%)
National	36.1 ± 0.6(35.0–37.1)^a^	10.3 ± 0.2(10.0–10.7)^a^	18.4 ± 0.3(17.9–18.9)^a^
Durango	37.6 ± 2.5(33.5–41.7)^a^	10.9 ± 1.1(9.2–12.6)^a^	20.2 ± 1.3(18.1–22.4)^a^
Lerdo city^1^	49.8 ± 3.6(42.8–55.1)^d^	18.4 ± 1.6(14.7–19.9)^c^	29.9 ± 2.9(24.4–33.1)^c^
Coahuila de Zaragoza	37.6 ± 3.3(32.1–43.1)^a^	12.3 ± 1.0(10.6–14.1)^b^	22.4 ± 1.4(20.1–24.7)^b^
San Pedro city^2^	48.6 ± 5.1(39.6–53.0)^d^	27.3 ± 1.7(15.9–33.8)^d^	34.9 ± 2.9(32.5–36.9)^d^
Other CERHA municipalities (moderate exposure, 9)	44.5 ± 2.5(42.2–48.6)^c^	17.0 ± 5.8(12.7–27.3)^c^	31.6 ± 6.4(28.1–42.9)^c^
Other CERHA municipalities (low exposure, 4)	44.2 ± 8.1(34.9–49.8)^c^	16.2 ± 2.3(13.8–18.4)^c^	27.7 ± 2.2(25.6–29.9)^c^
Non-CERHA municipalities (34)	40.2 ± 5.2(22.7–67.0)^b^	11.7 ± 1.7(7.5–17.1)^a,b^	21.7 ± 3.3(12.5–30.3)^b^

Multiple comparisons performed by Kruskal–Wallis test confirmed by Dunn's post-hoc test. Significant differences (*p < *0.05): a < b < c < d.

### Odd ratios by logistic regression model analysis

The associations between As exposure in drinking water and O&O, T2D, and AHT were analyzed by LMR, adjusting for sex (men and women), residence place (e.g., San Pedro, Lerdo and non-CERHA municipalities), exposition As level (e.g., high and low As exposure), and presence or absence of As in drinking water (e.g., CERHA and non-CERHA municipalities) (Table 7). The OD obtained by LMR is mathematically defined as the ratio between the probability of the metabolic disease in the exposed population to moderate or low As levels in drinking water and the probability of the metabolic disease in the non-exposed population in non-CERHA municipalities. Results evidenced that sex is a significant independent predictor of obesity, T2D, and AHT. For example, women are 2.3 times [inverse of OR and 95%CI 0.4 (0.2–0.7)] as likely to be obese compared to men independently of the residence municipality. On the other hand, men are two times more likely to be diagnosed with T2D [OR = 2.0 (1.4–2.3)] and AHT [OR = 2.0 (1.5–2.3)] than women.

**Table 7:** Logistic regression analysis for the association between As exposure in drinking water and obesity, T2D, and AHT adjusted by sex (male/female), exposure to As in drinking water (presence and absence), As level in drinking water (moderate, low, absence), and residence municipality (San Pedro, Lerdo, CERHA and non-CERHA municipalities, Durango, and Coahuila states).

Variable	Obesity		T2D		AHT	
	OR(95% CI)	Prob > χ^2^	OR(95% CI)	Prob > χ^2^	OR(95% CI)	Prob > χ^2^
Sex (Men vs. Women)	0.4(0.2–0.7)	0.005	2.0(1.4–2.3)	< 0.0001	2.0(1.5–2.3)	0.0240
As exposure (presence vs absence)	1.6(1.1–2.8)	0.009	1,5(1.1–2.7)	0.013	1.6(1.1–2.7)	0.012
As level (moderate vs. low)	1.0(0.9–1.2)	0.137	1.7(1.2–2.0)	0.02	1.8(1.7–1.9)	0.007
As level (moderate vs. absence)	1.5(0.8–2.9)	0.213	1.8(1.5–2.2)	< 0.0001	1.7(1.5–2.7)	< 0.0001
As level (low versus absence)	1.9(0.8–4.7)	0.17	1.5(1.1–2.0)	0.05	1.4(1.2–2.4)	0.032
CERHA municipalities versus Coahuila	1.3(1.2–1.5)	0.035	1.5(1.2–2.3)	0.01	1.6(1.5–2.3)	< 0.0001
CERHA municipalities versus Durango	1.3(1.2–1.4)	0.041	1.7(1.4–2.6)	< 0.0001	1.8(1.6–2.6)	< 0.0001
CERHA municipalities versus National	1.4(1.2–1.6)	0.032	1.8(1.3–3.1)	0.01	2.0(1.8–3.2)	< 0.0001
San Pedro versus National	1.7(1.2–1.9)	0.01	3.3(1.7–4.3)	< 0.0001	2.4(2.1–2.7)	< 0.0001
Lerdo versus . National	1.8(1.4–2.1)	< 0.0001	2.0(1.6–2.1)	< 0.0001	1.9(1.5–2.1)	< 0.0001

Prob > χ^2^: Probality > Chisq value (e.g., < 0.0001) indicates that ORs are significant.

Logistic regression analysis revealed that the residence municipality is a significant predictor of T2D and AHT. For example, San Pedro with moderate exposition level to As in drinking water presents a chance of 1.7 times greater to be diagnosed for T2D [OR = 1.7 (1.2–2.0)] and 1.8 for AHT [OR = 1.8 (1.7–1.9)] compared to Lerdo with low As exposition level. Also, the chances of being diagnosed with T2D and AHT were 1.5–1.8 and 1.4–1.7 times higher, respectively, in people inhabiting CERHA municipalities (presence of As in drinking water) than people in non-CERHA municipalities (absence of As in drinking water). The ODs calculated for CERHA municipalities, including San Pedro and Lerdo, compared to non-CERHA municipalities, in Coahuila and Durango states and nationwide, showed 1.5 to 3.3 and 1.6 to 2.4 times as likely to padece T2D and AHT, respectively. Regarding obesity, the CERHA municipalities (As presence, OR = 1.6 (1.1–2.8)) were 1.6 times higher than in non-CERHA municipalities in La Comarca province. Also, the chance to be obese were 1.3–1.9 higher in CERHA municipalities, including San Pedro and Lerdo, respect to Durango and Coahuila states and nationalwide. However, As level exposure contributed non-significatively to the obesity (1–1.9 times, Prob χ^2^ ≥ 0.137).

## Discussions

### Arsenic in the CERHA region in central-north Mexico

Ranges of As levels in groundwater representative of the CERHA region have varied from 0.5 to 880 µg L^−1^ in the existing historical dataset (1940s–2000s) to < 1 to 447.5 µg L^−1^ in 2005–2006 and 1.5 to 643 μg L^−1^ 2015–2017 surveys. Based on our results and the compilated database, As groundwater levels present a high spatial variability, with higher contents from the central-north towards the south of the aquifer (Tlahualilo-San Pedro-Viesca, the dirty zone) and a marked decrease towards the west of the aquifer (Torreon-Gomez Palacio-Lerdo, the clean zone). Similar spatial distribution was observed decades ago.

To mitigate the risk due to the presence of As in groundwater, the Rural Interstate System established a “polygon of clean water reservoir” to supply drinking water to > 130 villages and communities in the CERHA municipalities [Fig. 1;^23^]. However, the intensive extraction of groundwater, mainly from the clean water polygon in the metropolitan zone of Torreon-Gómez Palacio-Lerdo and its surroundings, progressively has caused the aquifer deficit (> 120–183 million m^3^ y^−1^) and groundwater depletion (> 1 to 3 m y^−1^) in the past decades^25^. Large-volume pumping creates unnatural groundwater gradients that mobilize the waters from the “dirty” (e.g., Francisco I Madero and San Pedro municipalities) to “clean” (e.g., Torreón and Lerdo municipalities) zone, promoting the intrusion of water with high contents of solutes, including As. The progressive groundwater depletion hypothetically increases the As levels because the pumped waters have interacted for a longer time with volcanic and intrusive rocks, one of the probable sources of As in the region. Consequently, the continuous movement and mixing of water masses from dirty to clean zones could increases the As levels in the clean water reservoir groundwater polygon. Given the severe health implications associated with exposure to As, it is imperative that a systematic and continuous monitoring program be implemented in the region.

In high-level CERHA municipalities, most wells showed As levels above the Mexican health standard for As in drinking water of 25 µg L^−1^^30^, a non-safeguard human health standard^31^ 2.5 times higher than the WHO recommendation. In low-level CERHA municipalities, most wells are below the Mexican health standard; however, > 80% of the analyzed wells had higher levels than the WHO guideline. In addition, practically all groundwater wells in the CERHA region are significantly enriched in As concerning typical values in natural waters of 1–2 μg L^−1^^10, 11, 32, 33^.

Regarding drinking water, to meet the Mexican health standard for As, the water operators in CERHA municipalities implemented a program called “tandeo” in which water concentrated in As (dirty) is mixed with low As water (clean) before being supplied to the population. For example, in San Pedro, As levels in groundwater are significantly higher than in drinking waters because of the tandeo. However, As levels in drinking water in San Pedro still averages four times the WHO guideline. In Lerdo city, groundwater and drinking water levels showed non-significant differences. Drinking water in Lerdo municipality met the Mexican As standard, 1.5–2 times higher than WHO guidelines. In San Pedro, the daily As uptake rate and cumulative exposure of As for men were two times higher than those for women and four times those for men and women in Lerdo city. Urinary As values were several times higher than in exposed than in non-exposed population.

The presence of As in groundwater and aquifer overexploitation impacts not only the population's health but also compromises the economic, social, and environmental sustainability of La Comarca Lagunera province^20^. People in CERHA municipalities are more vulnerable and have a poor living standard of life because the basic health services and sanitation are compromised.

Because the toxicological effects associated with prolonged exposure to As is drinking waters are very variable and can lead to severe skin damage (e.g., hyperkeratosis or hyperhidrosis), vascular and hematological lesions (anemia), neurological disorders, decreased sexual activity, malformations congenital and cancer (skin, lung, kidney, gallbladder)^8, 11, 15^, the WHO recommended a restrictive quality standard of 10 µg L^−1^ in drinking water^15, 31, 33^. Mexico maintained the previously WHO recommended limit in drinking water of 25 µg L^−1^ for several decades. Since May 2, 2023, the more stringent WHO quality standard of 10 µg L^−1^ has been mandatory in Mexico^34^.

### Metabolic diseases related to As exposure in drinking water

Epidemiologic literature provides evidence of As as metabolism disruptors inducing O&O^5, 35–38^, T2D^38–46^ and AHT^43, 47–50^. Research addressing the role of As in drinking water contributing to the prevalence of metabolic diseases such as O&O, T2D, and AHT is clear in people exposed to elevated levels. However, the long-term effects of low and moderate As exposure in drinking water and the risk of metabolic diseases remain unclear or controversial [e.g.,^4, 37, 40, 41, 51, 52^]. For example, while limited evidence suggests As exposure may be inversely related to BMI^53^, our findings do not support any association between BMI and As exposure in drinking water.

Besides, non-significant differences in obesity were observed between San Pedro and Lerdo municipalities with moderate and low As exposure levels in drinking water, respectively. However, based on the ORs between CERHA and non-CERHA municipalities, the chances for obesity in exposed people are 1.2–1.8 higher than in non-exposed people. The obesity prevalence is also higher in CERHA municipalities in the region than in the Durango and Coahuila states and national statistics. Another unexpected result is that women are > 2 times as likely to be obese than men, independent of the exposition As level or residence municipality.

Previous studies in CERHA municipalities of the La Comarca Lagunera province evidenced that inorganic As exposure may be diabetogenic^42^. The authors reported the ORs for T2D between 1.9 (1.1–3.4) and 2.7 (1.5–4.6) for groups with total urinary As of 64–104 μg L^−1^ and > 104 μg L^−1^, respectively. Our logistic regression model revealed that people in San Pedro (moderate As level in drinking water) showed more chances of being diagnosed with T2D regarding Lerdo municipality (low exposition level). Also, the chances for T2D in exposed people in CERHA municipalities are 1.5–3.3 higher for people in non-CERHA municipalities. Besides, the chances of being T2D diagnosed are double in men than in women. Thus, our prospective findings support an association of As exposure from drinking water with a higher risk of T2D in the range of levels observed.

The association of risks of coronary heart disease and stroke mortality with As in drinking water has been reported even in low levels of As (< 10 µg L^−1^)^39, 48^. In our study, ORs also evidenced that people in San Pedro showed a higher risk of AHT than in Lerdo. Besides, the chances for AHT in exposed people in CERHA municipalities are 1.6–2.4 times higher than in non-CERHA municipalities. Such as was observed for T2D, the chances for AHT are two times higher in men than women.

Men were more vulnerable than women to T2D and AHT diagnoses. Those results agree with the daily uptake rate and cumulative exposure of As higher in San Pedro than in Lerdo people and double for men than women. Regarding obesity prevalence, women showed higher chances than men and non-significant differences were observed between San Pedro and Lerdo with moderate and low exposure to As in drinking water, respectively. Thus, the relationships of As with obesity are unclear. The mean As concentrations for groundwater in non-CERHA municipalities are low (< 10 µg/L), compared with CERHA municipalities. However, the human health effects due to chronic exposure to very low concentrations of As through drinking water are uncertain.

### Metabolism of the As in exposed humans and association with metabolic diseases

Based on a systematic review of the literature, As metabolism in humans involves three main steps [e.g.,^19, 36, 54–57^]: (1) the ingested arsenate (AsV, much as 50–70%) or arsenite (AsIII) in drinking water is immediately absorbed from the gastrointestinal tract, (2) AsV is rapidly reduced to AsIII, and (3) by oxidative methylation, AsIII converse to mono (MAsIII), dimethylated (DMAsIII), or trimethylated (TMAsIII) metabolites.

Methylation facilitates the As excretion from the body, primarily in the urine (DMA >  > MMA > TMA). The degree of methylation varies with age (adults > children) and sex (women > men, especially during pregnancy)^54, 56^. Determining the As species in urine provides valuables insights into the transformation and metabolism of As within the body. Studies conducted in the exposed population of CERHA municipalities in La Comarca Lagunera province have reported high concentrations of As in urine, predominantly DMA (75–78%), followed by MMA (10–12%) and inorganic As (10–15%)^19, 55^.

The probable association between As and metabolic diseases is related to methylated arsenic. For instance, these compounds are potent disruptors of the pancreatic β-cells function and insulin production and inhibit the basal or insulin-stimulated glucose uptake by skeletal muscle, cultured adipocytes, or kidney cells^57^. Biochemical alterations related to the As metabolism in exposed humans can lead to various adverse effects. These include the induction of lipodystrophy and an increase in body fat content, fasting hyperglycemia, impaired glucose tolerance, insulin resistance, and disruptions in normal liver function and serum lipid profiles (e.g., total cholesterol, HDL cholesterol, LDL cholesterol and triglycerides)^35–37, 58^. The cumulative impact of these alterations contributes to the development of obesity and other metabolic diseases.

On the other hand, obesity and T2D may affect the body’s ability to metabolize As^36–38, 40, 42, 56, 58^, thereby potentially increasing or limiting its excretion through urine. The metabolism alteration is reflected in the strong correlation between urine As levels and creatinine, as observed in our study. In addition, elevated creatinine levels are observed in obese individuals and those with chronic kidney and renal diseases related to diabetes^59, 60^. The pathogenesis of T2D can affect the As metabolism and excretion. Fistly, T2D can result in increased urine production (polyuria) due to elevated glucose blood levels, leading to higher water elimination and greater excretion of certain substances, including As^40, 45, 61^. Conversely, advanced T2D accompanied by chronic kidney disease can cause decreased urine production, resulting in elevated levels of As in the body^43^. In our study, T2D-diagnosed participants and exposed to As in drinking water showed higher urinary excretion of As, and the levels of As in urine increased with the duration of diagnosis of T2D, suggesting a potential alteration in kidney function. Non-obese women excreted more As in urine, which may be explained by higher drinking water consumption.

Creatinine is commonly utilized to normalize urinary As levels in research studies^59, 62–64^. However, our investigation revealed a correlation between creatinine levels and urinary As levels, indicating that normalization may lead to inaccurate outcomes, such as an underestimation of As exposure^65, 66^. Additionally, we observed a correlation between creatinine levels and individual body weight, which is known to be influenced by muscle mass^66, 67^. Therefore, caution should be exercised when using creatinine as a normalization method for urinary As levels. Alternatively, urinary specific gravity, which is less affected body size and muscle mass, is an useful method for normalization instead of creatinine^68, 69^. In our study, this parameter was not measured.

While urinary As levels have been associated obesity, cardiovascular disease risk, and diabetes^19, 37, 42, 45^, strong associations have been found with As in drinking water and these health conditions^70, 71^. Our results provide evidence that using As levels in drinking water, including acceptable daily intake [ADI] and cumulative doses of As, is a a reliable method for assessing As exposure in the context of the research questions under investigation. However, determining the As species present both urine and water enables a more accurate risk assessment and comprehensive understanding of the potential health effects associated with As exposure, surpassing the limitations of solely measuring total As levels^19, 55^.Population living in CERHA municipalities are vulnerable not only to metabolic diseases (O&O, T2D, and AHT), such as our study evidenced, but also the association of As metabolism with cancer has been reported in La Comarca Lagunera. Mahlknecht et al. (2023)^20^ estimated that the lifetime risk of developing cancer for the population drinking water with As in CERHA municipalities ranged from 0.5 to 61 cases in 10,000 children and 0.2 to 33 cases in 10,000 adults^19^. The authors also reported that the probability of exceeding the acceptable incremental lifetime cancer risk level was 96% for children and 83% for adults.

### Additional routes of As to the population

The association between As in drinking water with total urinary As and As/creatinine evidenced that drinking water is the primary source of As in the study population. Therefore, in La Comarca Lagunera province, drinking water is the main route of As exposure. However, it is important to note that other sources of As, including dietary sources, should not be disregarded. For instance, large marine fish and seafood contain elevated levels of As and constitute a significant dietary As source for humans^72^. However, based on the declarations in the questionaries, the consumption of these seafood items is infrequent among the population (e.g., once a month with an intake rate < 5 g/day). Consequently, the contribution of marine fish and seafood to total urinary As is low in our study population.

In this CERHA region, groundwater is also used for irrigation in agriculture fields (cultivating alfalfa, walnuts, melon, and various vegetables) as well as for drinking by livestock (cattle, pigs, goats) and poultry (chicken and turkeys). The Comarca Lagunera is the leading national producer of dairy and poultry meat and a significant producers of fodder and walnut. Arsenic present in groundwater can enter the food chain, potentially contaminating agricultural and livestock products. Aditionally, tap water used in food preparation serves as a direct source of As for the populations in CERHA municipalities. Therefore, it is crucial to urgently investigate the probable presence of As in foods products originating from the region, as well as during their preparation in households within CERHA municipalities. This investigation should aim to assess the contribution of As intake from these sources, as well as its potential effects on the population. Furthermore, farm animals exposed to As through contaminated drinking water and feed materials could suffer adverse health effects^73^, highlighting the need for further investigation in this area.

## Conclusions

The naturally enriched As in groundwater in the CERHA region in central-northern Mexico has occurred since the 1960s (0.3–880 µg L^−1^) and persists in the present (0.5–447.5 µg L^−1^). Because of the water management policies in San Pedro and Lerdo cities, representative of moderate and low-level CERHA municipalities, the As levels in tap water decreased from 117.2–172.9 to < 70 µg L^−1^ and from 34.3 to 19.4–21 µg L^−1^ in the past two decades. However, San Pedro and Lerdo are 4 and 1.5–2 times higher than WHO guidelines regarding drinking water. Consequently, people are still exposed to low and moderate As levels in drinking water. Thus, As is still a health public concern. The high correlation between As in drinking water with total urinary As and As/creatinine and urinary As excretion and creatinine evidenced that drinking water is the primary source of As in our study population. The daily As uptake rate, cumulative exposure of As, total urinary As, and normalized values of As in urine to creatinine showed significant differences between sexes (men > women) and residence place (San Pedro > Lerdo > non-CERHA municipalities people).

In the province of La Comarca Lagunera, with low to moderate As levels in drinking water and epidemics of obesity, T2D, and AHT related to multiple causes, the quantification of As in drinking water as a risk factor for those metabolic diseases is complex. For example, non-significant differences were observed in obesity rates between San Pedro and Lerdo municipalities with moderate and low As level exposure, respectively. There was also non-significant association found between BMI and As exposure in drinking water. However, the odds of obesity were two times higher in exposed people (CERHA municipalities) comoared to non-exposed individuals (non-CERHA municipalities). Aditionally, people in San Pedro municipality showed odds of being diagnosed with T2D and AHT than people in Lerdo and non-CERHA municipalities. These finding provide further evidence that As exposure may increase the risk of developing diabetes and hypertension. Finally, odd ratios showed a higher risk of T2D and AHT for men than women, while women showed higher odds of being obese compared to men.

While non-indisputable evidence of cause and effect (e.g., As exposure and metabolic disease), our evidence is coherent with the higher prevalence of T2D and AHT related to As in drinking waters in CERHA municipalities. However, most people in the region are unaware of these acute adverse effects of As toxicity. Moreover, the prevalence of metabolic diseases is still increasing.

The inclusion of participants in same age ranges (45–64 years) and similar socioeconomic (e.g., access to health services, social security, basic services in housing, poverty rate, education level) and diet (e.g., portions of grains, vegetables, fruits and animal protein) was useful to control for differences other than As that can explain variations in metabolic diseases. However, results may not be generalizable to other population sectors. Therefore, future studies with a large sample size evaluating the differences among age categories and socioeconomic levels must confirm our findings. More epidemiological studies are needed on the effect of drinking water As exposure at different doses, on metabolic diseases risk.

Because, As from groundwater goes to the food chain, the presence of As in regional agricultural and livestock products as a second metalloid source to humans must be investigated. Also, the added As during food preoparation in homes related to tap water require be investigated. The trophic As transference and its presence in foodstuffs could also compromise the economic, social, and environmental sustainability of La Comarca Lagunera province.

To protect the human health of the As exposure to the La Comarca Lagunera population, the Mexican state must guarantee compliance with the human right to water and sanitation: (1) enough (20–50 L of drinking water per day), (2) accessible in homes or the vicinity, (3) healthy (without any health risk), and (4) available and accessible in all circumstances^74^. Therefore, the protection and management of water must be an absolute priority to supply free-As drinking water to CERHA municipalities. After decades of chronic exposition to As in drinking water, the Mexican state responds with the government action “Agua saludable para La Laguna” (Healthy water for La Laguna) to provide water in quantity and quality to the population by substituting As enriched groundwater from the aquifer with surface water from the Nazas River. Additional actions must include epidemiological studies to know the number of affected people to provide specialized medical attention to the effects of As on human health. To inform the population about the risk due the chronic exposure to inorganic As in drinking water and diet^75^. Also, to investigate the causes of the progressive lowering of the aquifer's groundwater levels, as well as the presence of As in this HACRE region.

## Supplementary Information

Supplementary Information.

## Data Availability

The authors confirm that the data supporting the findings of this study are available within the article and its supplementary materials.
