# Supplementary material for: Association of obesity, diabetes, and hypertension with arsenic in drinking water in the Comarca Lagunera province (north-central Mexico)
Source: Sci Rep. 2023 Jun 7;13:9244. doi: 10.1038/s41598-023-36166-5 (PMC10247736; doi:10.1038/s41598-023-36166-5)
Supplement: Supplementary file 1 — Supplementary Information. [file 41598_2023_36166_MOESM1_ESM.docx]

**Supplementary Information**

**Title: Association of obesity, diabetes, and hypertension with arsenic in drinking water in the Comarca Lagunera province (north-central Mexico)**

Short title: **Arsenic in drinking water and metabolic diseases**

Sánchez-Rodríguez B.L.^1^, Castillo-Maldonado I.^1^, Pedroza-Escobar D.^1^, Delgadillo-Guzmán D.^2^, Soto-Jiménez M.F.^3^*

^1^Department of Biochemistry, Biomedical Research Centre, Faculty of Medicine, Universidad Autonoma de Coahuila Unidad Torreon, Torreon, Mexico

^2^Department of Pharmacology, Faculty of Medicine, Universidad Autonoma de Coahuila Unidad Torreon, Torreon, Mexico

^3^Instituto de Ciencias del Mar y Limnología, Universidad Nacional Autónoma de Mexico Av. Joel Montes Camarena 82040, Mazatlán, Sinaloa, Mexico.

*Corresponding author. Email: [martin@ola.icmyl.unam.mx](mailto:martin@ola.icmyl.unam.mx); Telephone number: + 52 (669) 9852845; Fax number: +52 (669)9852613

**Table List**

Table S1. Demographic and socioeconomic characteristics in the selected municipalities in the La Comarca province in northern Mexico.

Table S2. Demographic and socioeconomic characteristics in the recruited participants classified by gender (women and men) and locations (San Pedro, Lerdo and non-CERHA municipalities.

Table S3. Main dietary components and portions self-reported by recruited participants by gender (women and men) and locations (San Pedro, Lerdo and non-CERHA municipalities).

Table S4. Source of drinking water, tap water or commercially purified (bottled), and daily ingestion rate (L d^-1^) for women and men from San Pedro, Lerdo and non-CERHA municipalities (Nazas, Cuencame, Simon Bolivar, and Mapimi).

Table S5. Consumption of alcohol and cigarette smoking habits as self-reported by the recruited participants classified by gender (women and men) and locations (San Pedro, Lerdo and non-CERHA municipalities. Percentages of the participants, number of cigarettes per day, and frequency of alcohol consumption per month.

Table S6. Physical activity self-reported by the recruited participants classified by gender (women and men) and locations (San Pedro, Lerdo and non-CERHA municipalities. Percentages of the participants.

Table S7. Characteristics of the participants in the study: anthropometric measures, systolic blood pressure (SBP, mm Hg), diastolic blood pressure (DBP, mm Hg), urinary creatinine (U-creat, µg L^-1^) urinary arsenic (U-As, µg L^-1^), and U-As/U-creat (µg g^-1^) classified by Diabetes, Hypertension, and Obesity diagnoses.

Table S1. Demographic and socioeconomic characteristics in the selected municipalities in the La Comarca province in northern Mexico.

| Municipality | San Pedro, Coahuila | Lerdo,  Durango | Cuencamé, Durango | Nazas, Durango | Mapimi, Durango | Simon Bolivar, Durango |
| --- | --- | --- | --- | --- | --- | --- |
| Population | 101,851 | 163,313 | 34,955 | 12,894 | 26,932 | 10,038 |
| Women | 51,902 | 82,457 | 17,440 | 6,369 | 13,462 | 5,057 |
| Men | 49,949 | 80,856 | 17,515 | 6,525 | 13,470 | 4,981 |
| Indicators of social deprivations (% population) | | | | | | |
| Educational backwardness | 17.9 | 13.9 | 16.1 | 21.1 | 20.8 | 22.6 |
| Access to health services | 32.2 | 22.0 | 26.6 | 16.7 | 21.5 | 24.2 |
| Access to social security | 46.9 | 42.4 | 56.7 | 76 | 51.2 | 69.8 |
| Quality and spaces in the house | 4.1 | 3.1 | 3.7 | 4.3 | 3.7 | 3.6 |
| Basic and quality housing services | 21.6 | 1.7 | 4.5 | 3.7 | 5.1 | 7.4 |
| Access to nutritious and quality food | 29.3 | 18.4 | 17.8 | 10.9 | 18.7 | 16.8 |
| Basic and quality housing services (% population) | | | | | | |
| Quality and spaces in the house | 4.1 | 3.1 | 4.5 | 4.3 | 3.7 | 3.6 |
| Dirt floors | 0.9 | 0.7 | 18 | 2.2 | 1 | 1.6 |
| Non durable roof materials | 0.3 | 0.8 | 3.7 | 0 | 0.1 | 0.1 |
| Non durable walls materials | 0.3 | 0.4 | 0.3 | 0.9 | 0.1 | 0.2 |
| Overcrowded homes | 4.9 | 4.1 | 4.7 | 6.7 | 5.3 | 5.5 |
| Basic services in housing | 21.6 | 1.7 | 4.5 | 3.7 | 5.1 | 7.4 |
| Non-access to water | 15.7 | 8.9 | 18 | 13.2 | 12.5 | 50.2 |
| Non-sewage drainage | 13.5 | 2 | 3.7 | 3.2 | 2.1 | 8 |
| Non-electricity | 0.2 | 0.2 | 0.3 | 0.3 | 0.2 | 0.2 |
| Non-chimney using firewood or charcoal for cooking | 1.3 | 0.7 | 0.7 | 1.8 | 0.1 | 0.9 |
| Condition of multidimensional poverty (% population) | | | | | | |
| Extreme poverty | 7.1 | 1.8 | 2.4 | 2.2 | 2 | 2.6 |
| Moderate poverty | 34.3 | 27.8 | 33.2 | 45.6 | 34.4 | 37.6 |
| Vulnerable by income | 9.2 | 10.3 | 8.7 | 5 | 10.4 | 5.8 |
| Vulnerable due to lack of basic services | 31.7 | 29.6 | 36.6 | 38.6 | 33.5 | 45.6 |
| Not poor and not vulnerable | 17.8 | 30.6 | 19 | 8.6 | 19.6 | 8.5 |

Dataset from the Report Annual of Multidimensional Poverty and Social Backwardness [1].

Table S2. Demographic and socioeconomic characteristics in the recruited participants classified by gender (women and men) and locations (San Pedro, Lerdo and non-CERHA municipalities.

|  | San Pedro | Lerdo | ^1^Non-CERHA |
| --- | --- | --- | --- |
| Participants (n= 257) | 77 | 110 | 70 |
| Women (n = 152) | 45 | 65 | 42 |
| Men (n = 105) | 32 | 45 | 28 |
| Age (years): Median(10-90th) | | | |
| Women | 52(45-63) | 54(45-64) | 57(45-63) |
| Men | 55(45-64) | 59(44-64) | 57(45-64) |
| ^2^Indicators of social deprivations (% population) | | | |
| Educational backwardness (<6 y) | 9.7 | 5.5 | 9.2 |
| Completed 8-12 y | 73.0 | 75.6 | 77.6 |
| Completed 8-12 y | 17.3 | 18.9 | 13.2 |
| Access to health services | 39.0 | 29.8 | 29.4 |
| Access to social security | 52.2 | 48.2 | 67.3 |
| Access to nutritious and quality food | 33.0 | 28.1 | 28.3 |
| Condition of multidimensional poverty (% population) | | | |
| Moderate poverty | 40.1 | 38.1 | 41.9 |
| Vulnerable due to lack of basic services | 40.9 | 31.4 | 39 |
| Other category | 19.0 | 30.5 | 19.1 |
| ^1^Nazas, Cuencame, Simon Bolivar, and Mapimi. ^2^Basic and quality housing services: 100% non-dirty floor houses, durable materials, and with basis services (water, sewage drainage and electricity). | | | |

Table S3. Main dietary components and portions per day, self-reported by recruited participants, categorized by gender (women and men) and locations (San Pedro, Lerdo and non-CERHA municipalities).

|  | Women | Men |
| --- | --- | --- |
| All cereals (maize, wheat, rice), and Leguminosae (beans, lentils) | | |
| San Pedro, Coahuila | 5(4-8) | 4(3-7) |
| Lerdo, Durango | 5(4-8) | 4(3-7) |
| Non-CERHA municipalities | 5(4-8) | 4(3-7) |
| All vegetables (tomato, green chili, onion, corn) and tubercules (potatoes, carrots) | | |
| San Pedro, Coahuila | 2(1-3) | 2(1-3) |
| Lerdo, Durango | 2(1-3) | 2(1-3) |
| Non-CERHA municipalities | 2(1-3) | 2(1-3) |
| All fruits (orange, banana, avocado, mango) | | |
| San Pedro, Coahuila | 1(0-2) | 1(0-2) |
| Lerdo, Durango | 1(0-2) | 1(0-3) |
| Non-CERHA municipalities | 1(0-2) | 1(0-3) |
| All dairy products (milk, cheese, yogurt). | | |
| San Pedro, Coahuila | 1(0-2) | 1(0-2) |
| Lerdo, Durango | 1(0-2) | 1(0-2) |
| Non-CERHA municipalities | 1(0-2) | 1(0-2) |
| All animal protein sources (eggs, meat, poultry, fish, and shellfish). | | |
| San Pedro, Coahuila | 2(1-3) | 2(1-4) |
| Lerdo, Durango | 2(1-3) | 2(1-4) |
| Non-CERHA municipalities | 2(1-3) | 2(1-4) |

Recommended portions [2-3]: All cereals 7-10 portions per day (tortilla and bread pieces, cup, cuff size), All vegetables 4-6 portions d^-1^ (cup), All fruits (fruit piece, cup) 2-4 portions d^-1^), All dairy products (240 mL glass, 30 g) 2-3 portions per day, All animal protein foods (90 g) 2-3 portions per day.

Table S4. Source of drinking water, tap water or commercially purified (bottled), and daily ingestion rate (L d^-1^) for women and men from San Pedro, Lerdo and non-CERHA municipalities (Nazas, Cuencame, Simon Bolivar, and Mapimi).

|  | *Drinking water source (%) | | Daily ingestion rate (L d^-1^) | |
| --- | --- | --- | --- | --- |
| Municipality | Tap water | Bottled | Women | Men |
| San Pedro, Coahuila | 30-45 | 55-70 | 1.2-1.8 | 1.6-2.2 |
| Lerdo, Durango | 65-77 | 33-45 | 1.3-1.9 | 1.8-2.3 |
| Non-CERHA municipalities | 70-77 | 23-30 | 1.2-1.9 | 1.6-2.4 |

* Most people declaring drinking purified waters also recognized the use of tap water for food preparation (e.g., rinsing and cooking food and dishwashing).

Table S5. Consumption of alcohol and cigarette smoking habits as self-reported by the recruited participants classified by gender (women and men) and locations (San Pedro, Lerdo and non-CERHA municipalities. Percentages of the participants, number of cigarettes per day, and frequency of alcohol consumption per month.

| Municipality | Habit | Women | Men |
| --- | --- | --- | --- |
|  | Smoking (yes, %) | Cigarettes d^-1^ | |
| San Pedro, Coahuila | 4.4-8.9 | 2.5(1-14) | 3(1.5-14) |
| Lerdo, Durango | 3.3-9.0 | 3.5(1-8) | 8(4-14) |
| Non-CERHA municipalities | 3.4-7.8 | 5(2-14) | 5(3-14) |
| Drinking alcohol (yes, %) | | Events month^-1^ | |
| San Pedro, Coahuila | 5.6-8.9 | 1(1-5) | 3(1-6) |
| Lerdo, Durango | 4.4-8.9 | 1(1-5) | 2(1-4) |
| Non-CERHA municipalities | 5.6-7.8 | 1(1-6) | 2(1-4) |

Table S6. Physical activity self-reported by the recruited participants classified by gender (women and men) and locations (San Pedro, Lerdo and non-CERHA municipalities. Percentages of the participants.

| Municipality | Sedentary | | Less than WHO recommendation | | WHO recommendation | |
| --- | --- | --- | --- | --- | --- | --- |
|  | Women | Men | Women | Men | Women | Men |
| San Pedro, Coahuila | 60.0 | 62.1 | 24.0 | 20.8 | 16.0 | 17.1 |
| Lerdo, Durango | 62.5 | 58.2 | 18.0 | 23.6 | 19.4 | 18.2 |
| Non-CERHA municipalities | 66.0 | 65.4 | 19.5 | 22.1 | 14.5 | 12.5 |

WHO recommendation for adults aged 18-64: at least 150 minutes of moderate-intensity aerobic physical activity throughout the week or at least 75 minutes of vigorous-intensity aerobic physical activity throughout the week.

Table S7. Characteristics of the participants in the study: anthropometric measures, systolic blood pressure (SBP, mm Hg), diastolic blood pressure (DBP, mm Hg), urinary creatinine (U-creat, µg L^-1^) urinary arsenic (U-As, µg L^-1^), and U-As/U-creat (µg g^-1^) classified by Diabetes, Hypertension, and Obesity diagnoses.

| Participant | Obesity | | | | T2D | | | AHT | |
| --- | --- | --- | --- | --- | --- | --- | --- | --- | --- |
| information | No | Yes | | | No | | Yes | No | Yes |
| Age (y) | 53.2±8.1 | | 50.8±7.3 | | 51.3±8.9 | | 53.3±8.8 | 49.4±8.3 | 55.6±8.9 |
| Weight (kg) | 66.9±10.6 | | 83.9±11.3 | | 74.2±12.1 | | 77.6±16.6 | 72.5±12.5 | 79.6±14.8 |
| Size (cm) | 159.2±9.7 | | 155.2±7.527 | | 156.8±8.8 | | 158.0±8.9 | 158.3±8.7 | 155.8±9.0 |
| BMI (kg m^-2^) | 26.3±2.4 | | 34.8±4.2 | | 30.2±4.5 | | 31.2±7.0 | 28.9±4.2 | 32.9±6.2 |
| SBP (mm Hg) | 125±17.8 | | 133±24.7 | | 128.9±21.4 | | 129.6±22.9 | 124.3±18.2 | 136±24.7 |
| DBP (mm Hg) | 76.9±9.2 | | 84.2±15.4 | | 80.5±14.6 | | 80.6±13.9 | 78.6±11.8 | 83.2±17.2 |
| FSG (mg dL^-1^) | 131±82.7 | | 112.9±59.5 | | 91.4±12.3 | | 180.0±99.3 | 123.6±82.2 | 119.5±56.0 |
| U-creat (µg L^-1^) | 1.0±0.37 | | 0.79±0.35 | | 0.77±0.29 | | 1.0±0.41 | 0.80±0.29 | 0.96±0.42 |
| U-As (µg L^-1^) | 17.6±22.7 | | 11.2±13.3 | | 11.5±13.6 | | 19.9±25.3 | 14.9±19.2 | 22.2±14.7 |
| U-As/U-creat (µg g^-1^) | 28.83±39.0 | | 20.63±17.6 | | 16.74±17.52 | | 20.77±17.0 | 19.6±31.85 | 25.2±18.0 |
| U-As/As water | 0.43±0.32 | | 0.45±0.58 | | 0.43±0.52 | | 0.45±0.35 | 0.41±0.31 | 0.47±0.46 |
| Drinking water (L d^-1^) | 1.7±1.0 | | 1.6±0.9 | | 1.6±0.9 | | 2.2±1.2 | 1.6±0.9 | 2.2±1.2 |
| Smoking (yes, %) | 7.8% | | 4.4% | | 8.9% | | 3.4% | 9.0% | 3.3% |
| Drinking alcohol (yes, %) | 7.8% | 5.6% | | 8.9% | | 4.4% | | 8.9% | 4.4% |

**Figure List**

Figure S1. a) Schematic representation of the case-control study. b) Flow chart of enrolment and recruited participants in the case-control study conducted during 2015 and 2017.

Figure S1. a) Schematic representation of the case-control study. b) Flow chart of enrolment and recruited participants in the case-control study conducted during 2015 and 2017.

**References**

1. Secretaría de Bienestar. 2020-2023. Informe anual sobre la situación de pobreza y rezago social 2022. Unidad de Planeación y Evaluación de Programas para el Desarrollo, Oficina de la Subsecretaría de Planeación, Evaluación y Desarrollo Regional. Accessed on https://www.gob.mx/ November 2022.

2. Pérez-Lizaur, A.B.; Palacios González, B.; Castro Becerra, A.L.; Flores Galicia, I. , 2014 Sistema Mexicano de Alimentos Equivalentes, 4th ed.; Fomento de Nutrición y Salud: Mexico City, Mexico.

3. Bonvecchio-Arenas, A.; Fernández-Gaxiola, A.C.; Plazas Belausteguigoitia, M.; Kaufer-Horwitz, M.; Pérez Lizaur, A.B.; Rivera Dommarco, J.Á. 2015. Guías Alimentarias y de Actividad Física En Contexto de Sobrepeso y Obesidad En La Población Mexicana; Intersistemas: Mexico City, Mexico, 126.
